# ‘But … Would I Be Able to Toast With Friends?’ When Service Users Ask for New Care Pathways

**DOI:** 10.1111/hex.14148

**Published:** 2024-09-05

**Authors:** Michele Rocelli, Ludovica Aquili, Arianna Palmieri, Diego Romaioli, Lea Ferrari, Elena Faccio

**Affiliations:** ^1^ Department of Philosophy, Sociology, Pedagogy and Applied Psychology (FISPPA) University of Padova Padova Italy

**Keywords:** personal care needs, proactive dialogue, self‐determination, standardised care, substance use

## Abstract

**Introduction:**

The WHO European Mental Health Action Plan (2013–2030) emphasises the need to generate services that are more inclusive and attentive to the co‐construction of care practices. This exploratory research investigates the needs of young substance abusers shown during their stay in residential communities; in particular, it explores the idea that treatment may include a new phase focused on how to manage moderate or controlled alcohol intake during residential care. Interviews with young ex‐users open up critical reflections on complete abstinence programmes from all substances, including alcohol, as a prerequisite for discharge and also provide examples of how to co‐design a plan for mindful drinking.

**Methods:**

Fourteen young adults, aged 19–32 years, non‐alcoholists, treated at rehab in Fermo, in central Italy, were interviewed during a programme between 6 and 18 months of period. They were asked about exploring needs in preparation for the conclusion of the rehabilitation pathway. From this exploration emerged the need to introduce controlled alcohol intake during the rehabilitation stay. This request became the focus of the semi‐structured interviews.

**Results:**

Three main themes emerged, which are as follows: (1) difficulties in integrating the new identity with the past of consumption, (2) resistance to the idea of total abstinence in social relations and (3) uncertainties about post‐community behaviour regarding alcohol intake. At the same time, three unexpected needs were expressed: (1) test the personal knowledge and skills on how to manage the alcohol intake, (2) receive support during the residential path to build up self‐control competence given the post‐discharge period and (3) build a personalised therapeutic path together with the supervisor and the operators while still at the rehab, according to the realistic lifestyle and routine outside the rehab.

**Conclusions:**

This research highlights the importance of personalising treatment based on each user's needs, going far beyond the standardised treatments for users previously considered unable of self‐control and self‐determination. For that purpose, the relationship between the users and the operators might be privileged, as it is able to cover the specific needs aimed for the new identity.

**Involving the Participants:**

The research sparked a discussion within the community, involving and initiating an open dialogue between the operators and the users, allowing them to focus on certain innovative strategies offered by the service, putting the users' needs at the very centre of the attention. The results were compared and discussed actively with the participants involved.

## 1 Introduction

Alcohol consumption is rooted and recognised as a habit of Western culture, which plays a key role in many social events, based on its symbolic and strongly connotative value of personal identity [1, 2, 3]. Different studies considered difficulties in people who wanted to reduce or quit alcohol intake [4]: Saying no to a drink could also mean saying no to the symbolic meanings connected to the social event, that is, conviviality, reciprocity and complicity with others [5]. Abstinence is recognised as a real problem in young adults, purely because they are forced to play a specific ‘active’ role, negotiate new rules in relationships and break up established role expectations [6]. Furthermore, the identity position of a non‐drinker can also be perceived as carrying stigma [7]; it must therefore be managed by the actor through practices of concealment, reporting and camouflage because he/she may appear as someone not able to relax and enjoy cheering the conviviality [4, 8, 9]. It could be an awkward situation where the non‐drinker has to hide, conceal or even explain the reason for abstaining, hence making the non‐drinker excluded from some social contexts [10].

Many studies show the double side of alcohol consumption: From one perspective, alcohol gives social opportunities, as explained earlier; from the other one, alcohol consumption could lead to abuse with negative consequences on behaviour, physical health and mental health. Much research in fact learned more about social and health consequences and risks that occur in alcohol abuse [11, 12, 13], including the pressure put on peers [14].

Especially where alcohol is considered a habit, adults could help interact and cooperate with young ones showing how to manage alcohol intake, to enhance positive social behaviour [15, 16].

Although many research studies [17, 18, 19] have proved that it is difficult to reduce or stop alcohol intake, only a few of them considered that the reintroduction of moderate consumption might have a positive impact on the post‐rehabilitation transition. This actual research aims to fill that gap.

### 1.1 Abstinence or Reducing the Damage? Different Approaches and Methods for Treatment

In the past, the main approach to cure alcoholism was based on total dry abstinence from alcohol, treating in fact the problem with dry abstinence not only as a cure but also as an aim. It has recently discovered a new approach based on reducing the damage [20]. This new approach is not aimed at abolishing alcohol at all, it is rather focused on decreasing the amount of alcohol intake or finding alternatives in how to consume it. The benefits that patients may ensue are not only mental and physical but also potentially they are encouraged to quit drinking drastically [21].

Studies have emphasised that many patients who settled to cut drinking progressively could decide to pursue quitting radically [22, 23].

Reducing the amount of alcohol intake does not mean giving a successful recovery pathway [24]; it rather means providing patients with time and resources, aiming to rebuild their lives and avoiding the imminent pressure as a result of a radical change.

Previous studies have documented the essence of the ‘historical dichotomy’ between the reduction of the damage and dry abstinence. Although in the past, these two approaches were considered incompatible, nowadays they can coexist alongside each other and can be blended in a more humanised and personalised care model [25, 26].

Both approaches are heading to constant development, along which there are multiple possible alternatives to follow. These alternatives include psychological and pharmacological supports, self‐aid such as Alcoholics Anonymous offering emotional support and helping to reinforce the motivation and Rational Recovery groups focused on regaining the self‐confidence [27, 28]. The motivational support showed that the most effective cure is aiming to reduce the alcohol intake [29]. Another effective cure that reached similar results is based on the cognitive‐behaviour approach, which helps the patients to learn how to handle contingencies, encourage positive behaviour and learn how to avoid risks and dangerous situations. The cure relies on rewarding patients with vouchers or another similar kind of compensation [30].

The pharmacologic therapy uses disulfiram, naltrexone and acamprosate to reduce craving drinking, often combined with the cognitive behaviour to enhance the results [31, 32].

Choosing the appropriate cure is a process to be personalised, depending on the needs and the circumstances of everyone [25, 26]. The path does not need to be linear and standardised, but it is necessary that the path has to be designed according to the patient's needs to support a healthier and satisfied life.

Although most recent therapy patterns progressively acknowledged how important it is to normalise alcohol intake and how consumer plays a role in society [33], there are still situations where abstinence is the only way to cure the disease. It is the case of young adults staying in residential rehabs, where stopping drinking completely is the only effective therapy to cure the addiction, as they are particularly vulnerable. However, for the rest of the population, prevention campaigns focus on reducing damages affected by alcohol dependency, being aware that the population would not abstain from alcohol and the culture of drinking will prevail. Young adults are usually prescribed dry abstinence, but it is worth considering that once they finish the therapy, they will be back to living in their familiar environment where alcohol is part of their culture, but without developing any skills of self‐control, and yet, young adults need to develop and test their skills on being a responsible when they drink.

Places like therapeutic communities (TCs) are vital to offer protected environments and stay away from temptations. However, they can slow down the process of social reintegration and consider dry abstinence as the most effective cure where relapse would be eventually a sign of enduring alcohol addiction.

This theory not only ignores the importance of how beneficial could be to learn how to drink moderately, but it could also avoid dependency and the belief that every kind of alcohol intake is a failure.

### 1.2 The Study's Aims

This research investigates the perceptions of users of a residential community for young substance abusers regarding the integration of strategies for moderate and controlled alcohol consumption into their treatment pathway. Particularly, the purpose is to investigate whether the inclusion of skills to manage alcohol consumption can facilitate a more successful transition into society after the completion of the residential programme. Users' perspectives on maintaining total abstinence as a criterion for discharge were also explored and some ideas for the co‐design of controlled alcohol consumption strategies within the community were brought into focus.

## 2 Materials and Methods

The research involved 14 people, aged 19–32 years, being treated in a rehabilitation centre in central Italy, non‐alcoholics, who were interviewed during their residential programme. The interviews involved participants who had been in the programme for a minimum of 6 months to a maximum of 18 months. The interviewer spent 12 months before conducting the interviews to improve familiarity with the residential context and service users.

These 14 people have been selected based on specific criteria: All of them had a history of substance abuse, but none of them had psychiatric conditions diagnosed or alcohol‐related disorders. Particularly, the rehab did not accept people with alcohol dependency, and, following this criteria, none of the selected people had significant alcohol‐related problems. This rehab offers a 6‐ to 18‐month residential programme, which is focused on social‐psychological and educational interventions to inform all about the consumption of the substance, constructively. Interventions include individual and group therapy sessions, training activities for the development of personal and social skills and constant ongoing support for social reintegration. The psychosocial interventions are designed not only to address the issues with substance consumption but also to improve the relation and communication skills of the participants. The community setting, which promotes both abstinence and controlled alcohol management, provides a protected but realistic environment to explore alcohol consumption behaviour safely.

All the participants have a history of drug abuse, without psychiatric conditions, or alcoholic disorders. They were in different stages of the therapeutic path: Half of the participants (7) started the therapy less than 3 months before, whereas the other half (7) started the therapy at least 3 months before; hence, the latter, with more autonomy using their phones, driving and going out the CT without any supervisions. Semi‐structured surveys were based on the research's purposes, which aimed to study four areas (Table 1).

**Table 1:** Research purposes and the areas of research explored throughout the semi‐structured interview.

Research purposes	Areas of research explored throughout the survey
Investigating the relationship with alcohol	Investigate how young adults describe their relationship with alcohol before and while they are in rehab, and the incentives that lead to the use of alcohol
Perception of abstinence and the moderate intake	Gather opinions about the feasibility and the effect on complete abstinence compared to the moderate intake of alcohol during the pathway rehab
Estimations of the risk from post‐treatment alcohol abuse	Explore the participants' estimations in how they can manage their control with alcohol once they have finished their pathway in the rehab, including the strategies and the tools they chose
The rehab community's role about controlling the alcohol intake	Investigate the user's feeling on how the CT about finding together the way about the cure during the rehab

The Ethics Committee of the School of Psychology of the University of Padua (4771) approved the informed consent about the research and shared it with each participant. The consent form was shown to each participant beforehand, read and discussed altogether, answering any possible doubts or questions.

### 2.1 Data Collection

Following a qualitative research approach, the data were collected through semi‐structured individual interviews, which allowed deeper and more personal discussions with each participant. These interviews were conducted in 1 year, from September 2021.

The data collection and analysis are based on the guidelines of the qualitative research [34]. We preferred the semi‐structured [17, 18] narrative interviews because these allowed us first to focus on the meanings and the topics we are interested in, and also to better understand other significant elements that could emerge during a conversation rather than a more schematic test. The interview in fact is considered not only a tool but also a method, because of the interaction process established between the interlocutors [17]. We realised one‐to‐one interviews into the rehab, all audio recorded.

### 2.2 Data Analysis

The collected interviews were audio recorded and transcribed, and then analysed, according to a constructionist point of view [35, 36]. The thematic analysis process was based on Braun and Clarke's [37, 38] guidelines, consisting of six different steps: get used to the data, generate the initial code, search the theme, review the theme, define and name the theme and produce the report. Each interview has been written and analysed to define and refine themes that emerge significantly, allowing the participants a thorough understanding of how to control and introduce themselves again to alcohol intake into the pathway of rehabilitation.

The aim was to learn how young adults [39] in the rehab consider alcohol intake and understand their expectations about reintroducing it in their lives in a responsible way and feeling about their rehabilitation path.

We shared the results with the participants in three final sessions, during which not only introduced the main results but also asked for feedback and thoughts about what their most important result was, and we are reporting in this article. The analysis that we have been through allowed us to understand specific meaning structures [38]. Through the analytic process, we identified, in particular, two thematic areas: The first is about issues associated with alcohol intake, in connection with how they juggle the new identity and the previous addicted one. Then, having a social life where alcohol intake is a habit and the participants fear not being able to control their intake. The second one is about the need to co‐design a path of rehabilitation together with the caregiver, to ensure a smooth and the easiest possible transition from rehab—where the end of the addiction is an almost compulsory step—to the step back to society.

## 3 Results

The results emphasise what young adults have been through during the rehabilitation path, especially when they go back to their own social lives and back to society again, in connection with alcohol intake as part of the socialising process, in opposition to their new identity of ex‐addicted of psychotropic‐psychoactive drugs.

Table 2 summarises the key results, which are explained in depth further down.

**Table 2:** Main results emerged from the topics based on the research aims.

Research purposes	Issues	Demands
Investigating the relationship with alcohol	Struggling to combine the new identity with the past time as abuser	Testing the own abilities to manage the relationship with the alcohol
Perception of abstinence and the moderate intake	Being hostile to lead on the idea of a complete abstinence in a social context	Being supported during the rehab and acquiring the self‐control to apply after being dismissed from the rehab
Estimations of the risk from post‐treatment alcohol abuse The rehab community's role about controlling the alcohol intake	Uncertainties about the post‐rehab attitude with the alcohol intake	Designing an ad hoc therapeutic project, together with the responsible and the operators, on the basis of each life experience

The analysis of this topic allowed us to understand if the ex‐abuser could drink alcohol, by playing the new role of an ex‐addicted.

### 3.1 Issues

#### 3.1.1 It Is Difficult to Combine the New Identity With the Old One

The hard challenge to make a new identity, born from the therapy, with the old one, lies in the idea of being an ex‐abuser, which can be fixed by working on the perception of the self, related to the intake of the substances. This step can be very complicated, and full of inner conflicts, as testified after one of the first breaks, during the first period of the rehabilitation path.

We started eating and I poured a beer, as time went by, I started to modify my perspective of drinking one beer only and, when the first glass was empty, I poured another one, and another one and so on. I didn't end up being drunk, but I was tipsy anyway and, in that instant, I realised that I was doing nothing wrong, I didn't exaggerate with my drinking, as normally I used to do with alcohol and any other random substances which you can get ‘high’. People around me were realising it and it was pleasant showing them I've changed my attitude. They've been drinking too. I couldn't understand how to combine the two things!

(P.8)

It is not easy, because as I am an addicted person, I know which process can trigger me at any time, the process which in the ‘body (the emotions n.d.r.) doesn't allow you to feel’ and from that moment you know that you can recognise immediately, but it is difficult to keep control of.

(P.12)

The aforementioned excerpts are very clear about the inner conflict between the parts of the self in contrast. The first excerpt shows how the participant started drinking one beer, with the purpose to keep control on the intake, but eventually he was struggling, ending up with topping the glass up many times. This behaviour not only shows the issue of keeping a moderate control but also the inner conflict between the intention to remain loyal to the new healthy lifestyle and to stay away from the temptation of falling in the old habits and previous identity as an ‘abuser’. It is important to see how the participant explains his approach, recognising it as a change in front of others, despite the awareness of a possible ‘slid’ (or relapse). The second participant (P.12) told what the purpose for him was using drugs before starting the rehabilitation path so that it was a matter of turning off the thoughts that were triggering the drug use, a mechanism that could have been triggered also with alcohol. He speaks about the strong consciousness of a relapse risk, describing ‘that mechanism as a sense of numbness for the body’, always there and always dangerous. It seems clear how precarious is the balance that the patients in the first phase of the rehabilitation have to keep.

Today, I decided to quit drinking, at least temporarily. I am about to start a new pathway where it will be discussed about different parts of myself. I really need to think straight in order to see well clear ahead what happens outside. When I will leave, I really need to feel what really happens to me, the feelings, the disease, everything, I want to be in touch with everything in order to work out the most of it. I think when you drink, you miss a lot.

(P.11)

By now, drinking has only one meaning to me: to share company. The meaning of drinking has changed to me right now. Of course, sometimes I got drunk. Someone tells me that this is a problem related to my previous addiction with substances, but I really don't know what it has to do with it, because when you finish with the rehab, you go out and see drunk people around … and they are not from rehab!!!

(P.7)

The aforementioned excerpts highlight two different points of view regarding the conviviality in social environment: Although some of the participants consider drinking a good reason to enjoy social life (P.7), others prefer dry abstinence deliberately to stay straight, to get a deeper and faster recovery (P.11). These different approaches show us a variety of strategies that participants want to combine the new and the old identities.

When I used to go out with my girlfriend, since the beginning I knew that that evening I would get drunk, not only because I felt uncomfortable to have sex sober with my girlfriend. I thought that using substances I wouldn't be up to it, I was scared ‘coming’ (to have orgasm) too early or didn't meet my girlfriend's expectations, also because I don't remember to have sex without having cocaine before, cocaine or any other substance or even Mdma.

(P.14)

The recovery process is much simpler compared to quitting the substance intake completely. It is quite a process with complicated compromises with the person's identity. The participants tell in their stories how difficult it is to pursue this path and how many problems involve personal achievements and the risks of failing, struggling and deep moments of introspection.

#### 3.1.2 Oppositions to the Idea of Total Dry Abstinence in the Social Environment

The opposition to the idea of dry abstinence in the social environment is complicated, with many different aspects to consider. It is not simply a result of craving to drink; it rather comes from a number of social demands and practical considerations wondering about society and cultural roles in which alcohol plays.

It is very difficult avoiding bars, as not to say it is impossible. As well as it is impossible not drinking in certain occasions like weddings. […] Rather than avoiding alcohol, it is worth it to understand the reason why alcohol is being used. Here, they teach us how to manage situations, moments, what could occur outside of here. If you are able to manage it independently you don't need alcohol, then you are the one who decides when and how much you can drink, isn't it.

(P.11)

If you get used to the abstinence from alcohol and afterwards you are in a situation where alcohol is so easily accessible, it is going to be a mess, the whole process will start again. While you are being able to manage yourself where the alcohol is accessible, here is the big challenge … honestly, out there, there are so many occasions where alcohol is at hand. Nowadays, alcohol is everywhere in Italy, so being able to measure it, to me it is necessary.

(M.1)

Dry abstinence is extremely difficult to keep along within peers' socialising environment. In this regard, abstinence may lead to isolation or social deviance as some of participants emphasised ‘antisocial’ feeling if sober. Furthermore, the control of alcohol intake is a question of making the own decision and being responsible, in similar situations. The attention shifts from avoiding alcohol at all—considered rather impossible—to learn how to behave over alcohol. This approach requires a great deal of awareness and self‐confidence in the own personal motivations, to resist the triggers leading towards the abuse.

If I force you, you don't do anything else but escape. But if you can get all the chances, then you are the one to choose. That is to say, you are the one who is responsible for what you are going to do, not the operator by telling you—this is forbidden, this not good, that is wrong. Instead, if you set to be free, the responsibility is in your hands, you are the one who have to choose what to do. When you are in charge of yourself, you care more.

(M.3)

How to decide whether moderate alcohol intake or prefer dry abstinence requires a great ability to balance social engagements with sober or self‐control, a sensitive equilibrium to keep, especially for people addicted to different substances.

Nowadays, in the world you feel an antisocial if you don't drink anything at all. This is crazy … what's wrong with drinking a beer with friends in the evening? There is nothing wrong about that.

(P.8)

It's not the fact that I don't drink, it's not the fact that I got ruined, but I have to stay always sober, I have to be well aware about what I am doing and do not trespass certain schemes. I didn't force myself, but I get on with it and I remain like so.

(P.14)

The opposition to the idea of being completely sober within the social relationship reflects not only self‐challenges but also pressures and demands from society itself. It is important then that the rehab approach takes these challenges into account, providing strategies and appropriate support in helping users to learn how to behave in the most effective and healthy possible way.

#### 3.1.3 Doubts About How to Behave Over Alcohol Intake, Once the Rehabilitation Path Is Completed

The uncertainty over alcohol intake after the rehabilitation path is a difficult and complex matter. This uncertainty reflects the expectation to maintain the progress built during the therapy, once back to a less monitored and more exposed to everyday life's temptations.

As soon as they said I would have gone to ‘verifica [1]’, I was excited and scared at the same time: excited because i would have been able to go home for a couple of days, after been in rehab for seven months, scared because I would have been back to my town, and I would have taken the risk to go back drinking. the morning I left I have clearly in my mind that I would have a beer at dinner, even though I knew I would have done something wrong.

(P.11)

One of the most significant aspects of this transition is the stress due to the awareness of the risk of relapse and the willingness to go back to the society environment, where alcohol is a sort of habit. The aforementioned excerpts explain this dilemma: On one side, the awareness of how to behave and how to manage the temptations/demands or other risks, acquired during the rehabilitation; on the other side, the situation had to be handled without any immediate support or the rehab's help. The uncertainty is quite clear over a self‐analysis after having drunk: The participants want to find the mechanism that led them to drink and understand the elements that dragged them beyond the limits prefixed. This is a vital process because it stimulates the capacity of self‐control and be more conscious about alcohol intake.

I always ask myself why I emphasise every time I drink. If I exaggerate, I ask to myself ‘Last night I set myself a certain limit which I didn't comply, why? What happened? The scenario was this, this, and that, so I could have felt inappropriate, and I could have wanted to overdo and decided to have another beer’. I ask myself more questions now (during this residential path n.d.r.) then before, but I don't know yet how I will manage once I will be out of the rehab.

(M.7)

Another relevant aspect is how the social pressure and the cultural expectations over the alcohol affect the participants' decisions. Drinking to adapt themselves to the social environment or to feel apart from the group has to be considered as an extra challenge to their efforts in being sober.

### 3.2 Needs

The participants at the beginning of the therapy and those in more advanced stages of the therapy shared three specific needs.

#### 3.2.1 Testing Their Ability in Handling Alcohol Intake

Testing and understanding the relationship with alcohol intake after the therapy is vital during the rehabilitation process and the social reintegration.

Alcohol can lead to drugs. If you know your limits, you can also understand how far you can go. Knowing that if you get heavily drunk, the next day you feel dirty because you abused alcohol, then you want to get high, that is the risk. You have to know how far you can get. If you know where you start from and where you finish, drinking within your limit, I can see nothing wrong with it because it doesn't imply anything else but just a bender.

(P.13)

The awareness of the possible consequences from trespassing the limits prefixed would be a risk of relapse, dragging in a loop to the past experience with addiction, is considered the main fear expressed as a risk to compromise the rehabilitation path already taken.

It has to get the experience to stay without it (the beer): when it is the one (the beer) which chooses you (the beer n.d.r.), you have to be able to say no, in order for you to nourish your defense and see how you feel without it (the beer). You can also tell me: ‘I don't like to stay without it (the beer)’, but you can't say it if you never even tried. It could be that you like being without it and you could also say: ‘I found many good things which I couldn't figure out while I've been drinking’, or you could also say: ‘I don't like staying sober and every Saturday I want to get drunk, I allow myself to do so, but not every day as a habit’.

(P.7)

Some of them chose dry abstinence for a certain period and then gradually monitored alcohol intake. This allows them to judge how alcohol affects their lives and if it is possible to enjoy a moderate alcohol intake without any risks. Others prefer to build their own criteria to define when the behaviour can cause problems or be acceptable.

Many stories told in this study urge us to design a personalised rehabilitation path over alcohol intake. The therapies have to follow specific steps and adjust the frame of periods according to each individual. Personalised programmes are needed, based on self‐consciousness allowing one to keep control of the own choices, rather than follow rules of limits randomly imposed.

I really would like to understand how much I can drink, in order to stop before getting ruined, because basically I never had a limit: I got high ruining myself, or I didn't drink at all. There are no compromises with me.

(P.5)

For many, the idea of being able to drink consciously is motivated to go back to normal life and belong to society. However, there is a friction between this reason and the urge to stay alert to avoid relapses. The need to test the ability to keep self‐control is difficult because it is a shift from participating in the rehab therapy to actually being the protagonist of the rehabilitation therapy. This process requires a subtle equilibrium between the interpersonal relation with alcohol of being sober and being conscious in society and the day‐to‐day lifestyle.

#### 3.2.2 Being Supported During the Rehabilitation Residency to Build a Resourceful Identity

I think it's right to have an alcohol‐free frame of time, so that partying without drinking; this will allow you to choose to drink or not. But you need to be escorted, otherwise you tell yourself only bullshit.

(P.11)

The stories reported earlier show that efficient support would be not only based on strict and standardised cures but rather on personalised and tailored therapies according to individual experiences. The idea of being assisted is vital because it eases the transition transforming an automatic behaviour to a conscious and thoughtful choice.

Sometimes you know that drinking is wrong and not drinking is good! Of course, it is like that, I am a smoker, and I know perfectly what it's like! However, one thing is if you tell me that smoking kills you and something else is if I have someone who leads me to try to reduce or even to quit smoking … in the same way works with alcohol … you have to find your way.

(P.2)

The user is motivated to recognise and find a way for a compatible and realistic alcohol intake. The process implies, in a way, to dismiss impositions or to ‘inherit’ identities, and pursue to rebuild the self.

If you are escorted by someone who tells what has to be done, that makes the difference. If the operator orders me not to drink, regardless … what do you think I can learn? I learned what is right for the operator. Though, if the operator allows you to find a way together with you, the perspectives are different then.

(P.11)

This need has a specific connection to the operator who plays the role throughout the rehabilitation. In this case, the operator is a guide or a mate throughout the rehabilitation path, avoiding imposing rules or limits randomly. The operator is rather someone who ‘escorts’, supports and understands the user throughout the experiences, mistakes and lessons learned, in a friendly and specifically tailored environment. This rational approach is a result of an extremely efficient treatment demand, allowing the users to compare their own personal position in front of problems faced during the exclusive and personalised rehabilitation path.

I would be able to drink when I will make a link between my mind and my body when I will live my feelings, but it is something that I can't do by myself … I am here to understand with them what I need to do.

(P.5)

‘Live my feelings’ and link ‘my mind and my body’ are very important aspects to be considered during the rehabilitation path, because it creates the opportunity to approach the addiction topic from different points of view, certainly alongside the therapeutic one.

#### 3.2.3 Co‐Designing Together With the Rehab Manager and the Operators Definitely Results to Be an Organic Therapeutic Path From the Practicality of the Real‐Life Point of View

So far, I don't say to myself ‘I should or I should not drink’, I see myself in a hypothetical future when I will be able to manage my feelings, and my emotions; I have to recognise my embarrassment, and when you accept that, then you don't need someone to lead you the way. Only then, yes, I can treat myself with a glass of wine.

(P.14)

The aforementioned excerpt clearly demonstrates the need to set up a specific therapy, aimed to cover and plan ahead the expectations for future everyday life. The ability to deal with feelings and emotions in a practical way, rather than avoid or even suppress them with alcohol intake, is the main goal here. The future perspective to learn ‘how to deal with my feelings and my emotions’ means certainly finding a long‐term solution, rather than a temporary or even an inappropriate one. The useful path here is where it is possible to let the user play an active role, as a protagonist, as the main character of its own rehabilitation path. The residential rehab environment can be useful for training and to practise with a sense of self‐awareness and self‐control, to be ready to use it in society at the end of the rehabilitation path. This is the message that many participants declared, also expressing the urge to be challenged by reintroducing the alcohol intake in social life. The active role mentioned earlier here is the support operators and therapists in fact urged in pursuing this experimental path, focused on working on a personalised therapy and the users' self. The participants are urged to find spaces and moments to think things through, to allow them to enforce a sense of self‐awareness and responsibility; hence, the team will cooperate guiding and helping them to find those spaces.

In here (in rehab), going out and coming back drunk is considered bad, not a relapse. To me this is disrespectful, first of all to the community who lives in the rehab and also in respect to the position you engaged in the rehab, replacing someone else in need to have that place in the rehab. If you want to do something like that (getting drunk) you sign out, and you do your life, you go back home. Nobody is forced to be here, if you stay here you have to follow the rules.

(P.1)

The resource of dealing with alcohol intake, instead of avoiding alcohol intake at all, adopted in the rehabilitation rehabs, could be extremely valuable when it is aimed to involve everyone's sense of responsibility and not the individual's only. The decision to drink could have a dangerous impact on the most vulnerable ones. The awareness and responsibility towards others, senses on which the rehabs generally work thoroughly, are profitable for the rehabilitation paths because it allows to work on sharing responsibilities at different levels and on respecting others.

## 4 Discussion

The evidence gathered earlier for this research highlights that the participants' preferred approach is the one based on the reduction of the damage. The alcohol intake during social events has been identified as the main challenge for young adults to achieve, once finish the rehabilitation path. Aligned with the other research studies [1, 2, 3], the participants have an ambivalent attitude towards alcohol: The social life and the symbolic value within the Western society make it impossible to remove alcohol completely from everyday life. In contrast, the participants want to be supported in reintroducing alcohol, as this is a process where the identity has to be rebuilt in some ways. Nobody, in fact, aimed abstinence as the main goal; it has been considered as a tool instead to build boundaries and to manage attitudes towards alcohol, based on personal experience. The young adults emphasised that they need a different approach to cure the addiction, not only innovative and personalised but also particularly focused on building the rehabilitation path up together, during the period on the residential rehab. This arrangement of cooperation, in fact, wants to cover the discrepancies between the real needs of the users and the standard methods, to simplify every single specific need and promote a setting up of new effective and realistic strategies. Making the users part of the rehabilitation process is essential to manage every challenge linked to the alcohol intake within the social environment.

However, the actual scenario has significant risks, causing in fact accidental situations with negative behaviours and consequences where alcohol could be felt legitimate during the rehab residencies. This could be a big risk because it could divert the main purpose of the therapy to a misjudged approval of alcohol or any other substance intake. Although to avoid unfavourable effects, it has to take into account that, besides considering each young adult's needs, we also have to consider the reason why they were incapable of managing the intakes appropriately so much that they needed rehab. Also, there are big issues juggling situations where, in the same residential rehab, someone can drink and someone else can't, because the diagnosis of addiction makes that one completely abstinent.

Nevertheless, the rehabs are settled in little mini hub societies, a reflection of the external structure, where users with different needs and alcohol limitations live together: Some of them want to drink, others just cannot drink, others need guidance and need to be trained to learn how to drink in a responsible way.

This cooperative approach explained earlier offers many resources: First of all, it allows us to deeply understand how young adults think and what is their perspective in facing problems, otherwise these problems tend to reside in their minds and never revealed; it will then be much easier to design more realistic therapies. This method appears to open the way to a proactive dialogue between users and operators, talking about problems that are needed to be cared for ‘first‐hand’, in a ‘supervised’ therapeutic environment, based on cooperation and reciprocity. It is vital then to find a perfect equilibrium between tailoring and structuring the therapeutic path. Through this equilibrium only, we can be certain that the therapeutic path will be successful and secure, covering the users’ needs, without jeopardising the standards of the therapies and the safety in the residential rehabs.

## 5 Conclusions

Research on public health innovation considers the vital importance of the collaboration between the health team and the users, to address more complex demands [40]. Expressions such as co‐production [41] and co‐creation [42, 43] represent this relation. A crucial aspect is how to open a more frank and direct dialogue between operators and users, involving the users' network of friends and family so that the users become the main protagonists of therapeutic decisions [44]. The literature highlights the need to equalise the interaction between the healthcare staff and the consumers, without hindering the relationship [45]. However, the experiences of involvement remain limited, especially in the context of substance consumption [46].

This research's results show that it is important to tailor treatments to the users' needs, working out new treatments, much more effective than the standardised ones, where the users are considered incapable of self‐determination and self‐control. Through this constructive dialogue between the users and the operators, it was possible to identify specific demands related to the change of the identity process, resulting in an innovative and effective incubation of ideas. The results obtained reinforce the importance of an ecological and personalised therapy that takes into account real‐life situations, facilitating a more effective and conscious social reintegration.

The research highlighted not only the complexity but also the relevance of the matter of alcohol intake, a current topic about which the experts in the field are constantly debating. Allowing to drink or not young adult with group rehab is not easy at all, particularly it urges the priority to investigate thoroughly this matter adequately. It is relevant to consider that the interviews enabled the young adults to open their dialogues with their caregivers. This vital step is often fading away because of the standardised care processes, the roles played and the not‐shared personal aims pursued independently; it is finally a significant step that represents an important milestone in this kind of therapy.

## 6 Clinical Implications

The research explained earlier contributes to the studies about the identity and its resilience when substance abuse occurs, with a different approach to alcohol intake. The research provides useful results to the experts in the field of drug addiction and to people and families involved in this kind of situation. From a clinical theoretical point of view, the research questions the complete abstinence approach for each member of the rehab. The research in fact disapproves of the idea, established in the studies conducted so far, that an addicted person is considered incontinent, or someone unable to self‐control, especially within the society environment, for example, where the alcohol intake could be triggered. From a clinical practical point of view, our research suggests the benefits granted from co‐design [47] controlled drinking experiences within the rehab residencies, offering the users the chance to express themselves and highlight their experiences/beliefs. This is helpful to ‘learn how to drink well and drink bad’, then reaching only positive results at their return to society, after the end of the journey in the rehab. Ultimately, the research offers the caregivers to play a new role, not forcing and correcting [48] but rather guiding and empowering young adults to cooperate with ‘new coaches’ [49, 50, 51, 52], helping them to develop self‐control abilities.

## 7 Limitations

The study focused on a limited number of participants within a single residential setting, which may affect the generalisability of the findings. Moreover, the exclusive involvement of participants whose treatment occurred in a single TC restricts the applicability of the results to other contexts.

One of the limits of this research is that we couldn't interview more people from other rehabilitation residencies. However, we think that the limit matches with the resource [53, 54]. We believe in fact that the results could not be aligned with other rehabilitation residencies because our participants are in an environment where they are responsible and get involved in the co‐design of the rehabilitation path.

Future research could aim to enhance user engagement beyond the traditional paradigms of ‘consumption’ or ‘abstinence’. Investigating how various approaches may either promote or hinder positive outcomes in rehabilitation could provide valuable insights [52, 55, 56].

## Author Contributions

**Michele Rocelli:** conceptualisation, methodology, data curation, writing–review and editing, writing–original draft, formal analysis, investigation. **Ludovica Aquili:** methodology, writing–review and editing, formal analysis, data curation, writing–original draft. **Arianna Palmieri:** writing–review and editing, validation. **Diego Romaioli:** validation, writing–review and editing. **Lea Ferrari:** validation, writing–review and editing. **Elena Faccio:** conceptualisation, methodology, funding acquisition, writing–original draft, writing–review and editing, formal analysis, data curation, supervision.

## Ethics Statement

Approval for the study was obtained from the Ethical Committee of the School of Padova at the University of Padova (Approval No. 4771). All patients provided written informed consent before enrolment in the study.

## Consent

The participants were informed of their rights to withdraw at any time. They signed a written informed consent form regarding their participation in the research and its publication.

## Conflicts of Interest

The authors declare no conflicts of interest.

## Data Availability

The data that support the findings of this study are available on request from the corresponding author. The data are not publicly available due to privacy or ethical restrictions.
